# Cyclic Comonomers for the Synthesis of Carboxylic Acid and Amine Functionalized Poly(l-Lactic Acid)

**DOI:** 10.3390/molecules20034764

**Published:** 2015-03-16

**Authors:** Markus Heiny, V. Prasad Shastri

**Affiliations:** 1 Institute for Macromolecular Chemistry, University of Freiburg, Stefan-Meier Str. 31, Freiburg 79104, Germany; E-Mail: markus.heiny@makro.uni-freiburg.de; 2 BIOSS Centre for Biological Signalling Studies, University of Freiburg, Schänzlestr. 18, Freiburg 79104, Germany

**Keywords:** poly(lactic acid), copolymerization, α,ω-epoxyesters, carboxylic acid, amine

## Abstract

Degradable aliphatic polyesters such as poly(lactic acid) are widely used in biomedical applications, however, they lack functional moieties along the polymer backbone that are amenable for functionalization reactions or could be the basis for interactions with biological systems. Here we present a straightforward route for the synthesis of functional α-ω epoxyesters as comonomers for lactide polymerization. Salient features of these highly functionalized epoxides are versatility in functionality and a short synthetic route of less than four steps. The α-ω epoxyesters presented serve as a means to introduce carboxylic acid and amine functional groups into poly(lactic acid) polymers via ring-opening copolymerization.

## 1. Introduction

Over the last few decades, biocompatible polymers have become firmly established in the biomedical sciences due to their wide range of applications such as micro- and nanoparticulate drug delivery systems [1,2], scaffolds for tissue engineering [3,4,5] and coating applications [6,7]. Furthermore, the shift from biostable to biodegradable polymers, in medical as well as packaging and “green” applications [8], has led to a surge in research on certain polymer classes with poly(α-hydroxy acids) (PHAs) being prominent examples. Poly(lactic acid) (PLA) as the most important of the PHAs is derived from 100% renewable resources and has emerged as early as the 1970s as a material for degradable surgical sutures [9]. Since then it has been extensively explored for further uses in the medical arena such as drug delivery systems [10,11,12] and orthopaedic devices [13], often as one component in copolymers, e.g., with poly(ethylene glycol) [14] or poly(glycolic acid) [15], another important representative of the PHA class.

However, PLA and the other PHAs in equal measure are unsuitable for applications in which the presentation of biological motifs is a key requirement in assuring the desired cell responses [16]. They suffer from the absence of any functional moieties along the polyester backbone that are amenable for a functionalization of the materials in order to exploit a wider array of applications depending on those kinds of interactions between the materials and biological systems. Thus the functionalization of PLA is an active area of research and numerous routes have been developed to achieve this goal [17]. Among those are the usage of functional initiators, resulting in chain-end functionalized polymers [18], and post-polymerization procedures such as hydrolysis and aminolysis reactions [19,20]. Whereas in the former method the extent of functionalization is limited to a single moiety at the chain end, the latter suffers, in many cases, from severe polymer-chain degradation in the course of the reaction. Therefore, copolymerization of lactide (LA) with functional monomers represents a more suitable approach for the introduction of functional groups into the polymer backbone. In this context, the work of Vert on the copolymerization of LA with functional lactones [21] has set in motion the employment of various (protected) functional cyclic compounds in ring-opening polymerizations (ROPs) with LA. Examples are early works on functional glycolides [22], the synthesis and application of functional lactides [23] as well as functional esteramides (depsipeptides) [24]. Major drawbacks of many of these approaches are the complicated and often low-yielding synthetic routes towards the functional comonomers.

To address this constraint, we have developed a versatile approach towards the functionalization of polyesters employing functional α,ω-epoxyesters [25]. The epoxy moiety in these compounds can undergo ring-opening in the presence of organometallic catalysts such as tin(II) ethylhexanoate and they are therefore well suited as comonomers in the ROP with LA. The main advantage, however, is the straightforwardness with which the epoxyesters can be functionalized prior to the ROP. It is possible to introduce a wide array of functional groups, encompassing groups that are amenable to post-polymerization reactions, via an easy esterification reaction.

In this work, we present the synthesis of α,ω-epoxyesters functionalized with protected carboxylic acid and amine groups for the ROP with LA and investigate their potential for the incorporation of these valuable functionalities into PLA polymers.

## 2. Results and Discussion

### 2.1. Monomer Synthesis

**Scheme 1:**
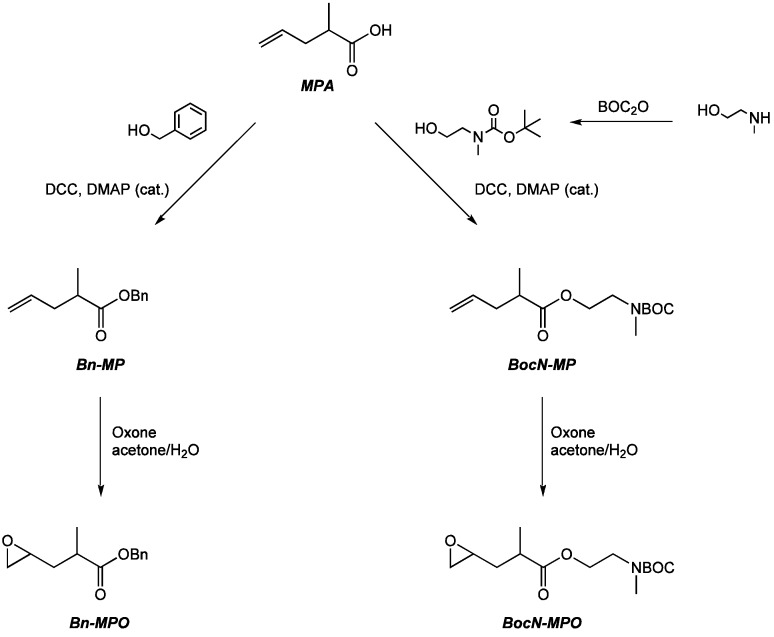
Syntheses of functional α,ω-epoxyesters from MPA.

Functional α,ω-epoxyesters monomers that could be applied as comonomers in the ROP of LA were prepared in straightforward two- and three-step syntheses. In detail, MPA was reacted with benzyl alcohol in an esterification reaction using DCC and catalytic amounts of DMAP, followed by an Oxone epoxidation to yield benzyl 2-methyl-3-(oxiran-2-yl)propanoate (Bn-MPO). 2-((*tert*-butoxy-carbonyl)(methyl)amino)ethyl 2-methyl-3-(oxiran-2-yl)propanoate (BocN-MPO), the comonomer for the introduction of protected amine functionalities, was synthesized in an analog fashion with the difference of an additional step to generate the BOC-protected 2-(methylamino)ethanol (Scheme 1).

**Figure 1:**
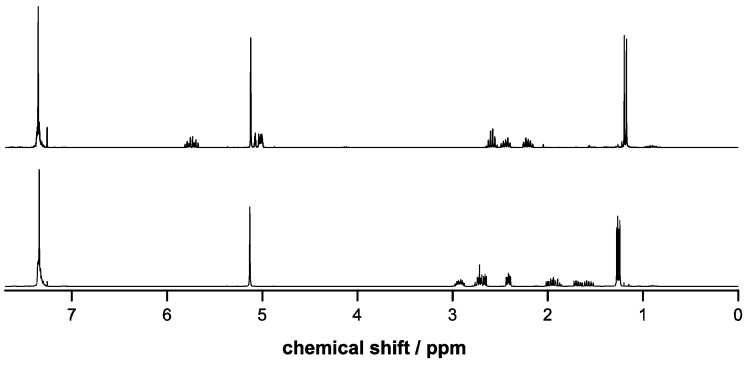
^1^H-NMR spectra of Bn-MP (top) and Bn-MPO (bottom).

**Figure 2:**
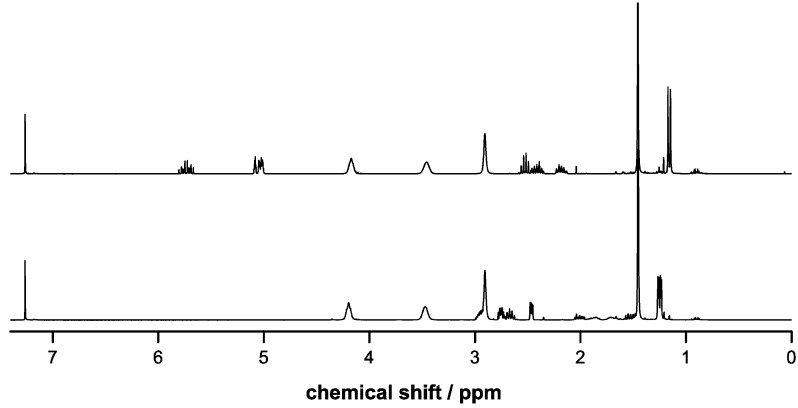
^1^H-NMR spectra of BocN-MP (top) and BocN-MPO (bottom).

The esterifications were achieved under mild conditions at low temperatures in quantitative yields and high purities as confirmed by ^1^H-NMR spectroscopy (Figure 1 and Figure 2) and elemental analysis. Trace amounts of DCU byproduct were removed after the epoxidation step by flash chromatography. The conversions to the epoxides using Oxone in acetone/H_2_O proceeded with yields around 50% in both cases with the possibility to fully recover the unreacted alkene reagents Bn-MP and BocN-MP by flash chromatography. The conditions of the epoxidation reactions with temperatures below 5 °C and buffered pH between 7.2 and 7.5 were sufficiently mild to preserve the previously formed ester bond.

### 2.2. Copolymerizations of Bn-MPO and BocN-MPO with LA

**Scheme 2:**
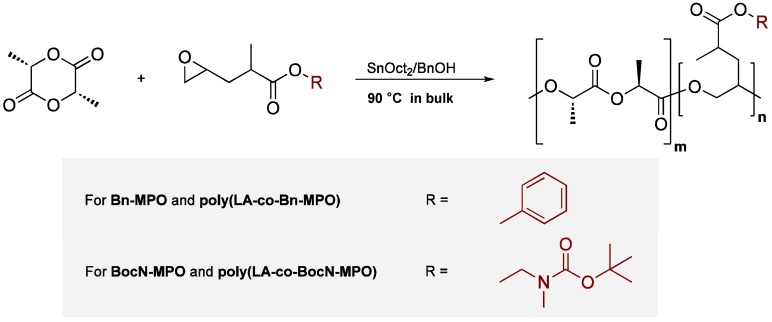
General scheme for the syntheses of PLAs functionalized with Bn-MPO and BocN-MPO.

The copolymerization reactions of the comonomers with LA were optimized with regards to molecular weight and polydispersity as well as the efficiency of the comonomer incorporation. For that purpose, ring-opening copolymerizations of the two monomers with LA were carried out, applying SnOct_2_ with BnOH as co-initiator in bulk reactions (Scheme 2).

**Figure 3:**
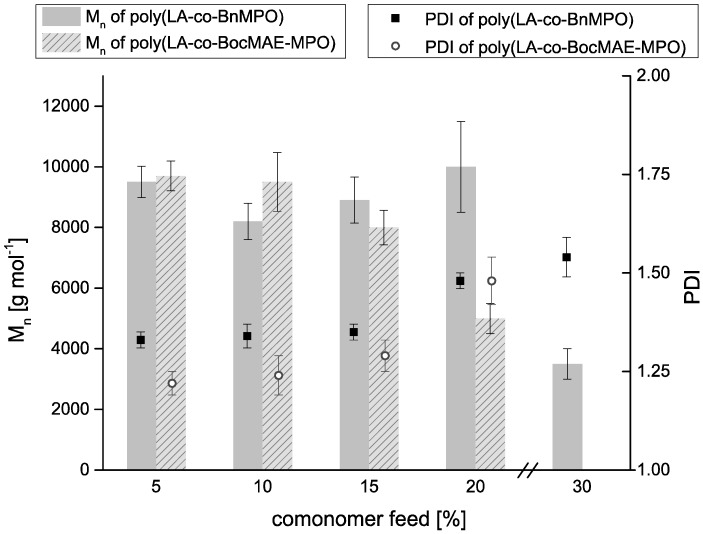
M_n_ (columns) and PDI (squares and circles) of synthesized copolymers dependent on comonomer feed.

Polymerization conditions were chosen as to yield polymers with number average molecular weights (M_n_) of around 10,000 g·mol^−1^. This molecular weight range has been proven suitable for the generation of nanoparticles via the nanoprecipitation method [26,27], an application that might prove highly interesting for the presented copolymers. Monomer molar ratios in the polymerization feeds were varied from 5% to 30%. Previous findings have indicated that for the polymerization of α,ω-epoxyesters with cyclic lactones (namely ε-caprolactone) and the aforementioned initiating system, reaction temperatures of 90 °C are favourable for obtaining polymers with expected molecular weights and molecular weight distributions [28]. It was found that for the system described here, polymerization temperatures of 90 °C using SnOct_2_ and BnOH amounts of 0.7 mol % and 1.4 mol %, respectively, were optimal to yield copolymers with M_n_ in the range of the value aimed at (Figure 3). The PDI of the obtained polymers was slightly higher with increased comonomer feed yet was with values between 1.3 and 1.6 for all comonomer feeds within the expected range of ring-opening polymerizations applying tin compounds as the initiating system [29]. This signifies that all polymers exhibit a relatively narrow weight distribution without the occurrence of lower molecular weight species. The involvement of the ester side groups in transesterification reactions possibly leading to branching of the copolymers cannot be excluded. However, considering the low polymerization temperature of 90 °C and the moderate reaction time of 12 h, these effects are believed to be of only minor significance as has been investigated for the applied catalyst SnOct_2_ [30].

One has to note, however, that in the case of the BocN‑MPO comonomer M_n_ was distinctly decreased with increased comonomer feed in the polymerization mixture. Increasing the comonomer feed from 5% to 20% resulted in a reduction of the molecular weight by half. The polymerization product obtained with a BocN‑MPO feed of 30% was an oily substance showing values of M_n_ below 1000 g·mol^−1^. In contrast to that, Bn-MPO incorporation only affected the molecular weight notably at high comonomer feeds of 30%. This is contrary to our previous findings on copolymerization of α,ω-epoxyester with LA and ε-caprolactone in which it was found that the epoxide comonomers had a severe impact on the molecular weight of the copolymers [28,31]. The observed differences in the extent how the introduction of the two comonomers affects the molecular weight may be due to various reasons such as monomer stability or the occurrence of side reactions. A possible explanation could be the presence of considerable amounts of aromatic moieties in the polymerization mixture due to the benzyl ester protecting group of the Bn-MPO comonomer. It has been shown that the presence of compounds such as styrene or α-methylstyrene in polymerization procedures of LA with SnOct_2_ can lead to a suppression of transesterification reactions due to interactions of the aromatic compounds with the metal center of the catalyst [32]. The absence of intramolecular transesterification reactions (back-biting) that lead, especially at high temperatures and long reaction times, to a decrease in molecular weight of the formed polymers, could be the reason for the observed differences in molecular weight evolution of the two copolymers. As to why for a Bn-MPO feed ratio of 30% a pronounced drop in M_n_ was observed is not readily explained and might be due to an increased extent of unfavorable side reactions at high comonomer feed ratios.

### 2.3. Incorporation of Comonomers

The comonomers Bn-MPO and BocN-MPO were examined for their potential to be incorporated into the polylactide backbone. Copolymerization reactions were carried out using varied comonomer feeds from 5% to 30%. The extent of comonomer incorporation into the polymer backbone was estimated by ^1^H-NMR.

**Figure 4:**
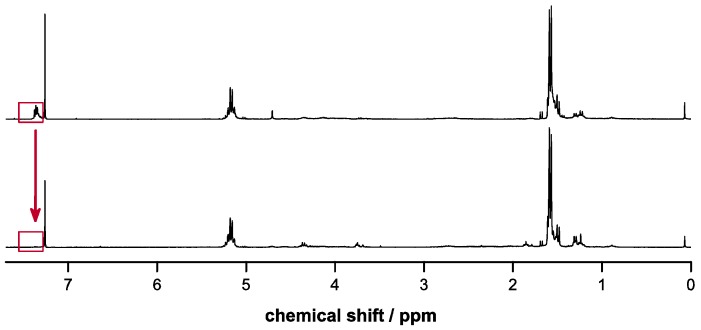
^1^H-NMR spectra of poly(LA-co-Bn-MPO) (above) and the respective deprotected copolymer (below) with the signal used to estimate comonomer incorporation highlighted in red.

**Figure 5:**
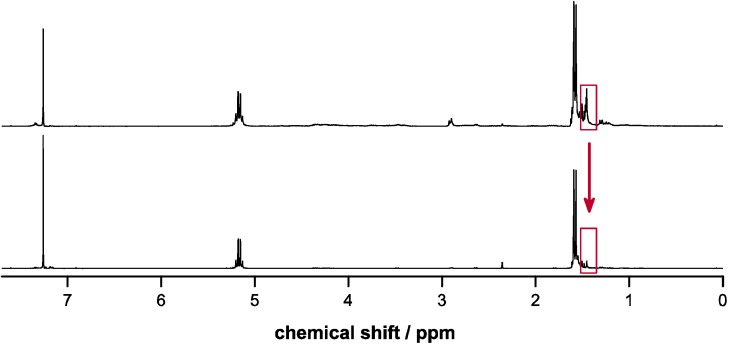
^1^H-NMR spectra of poly(LA-co-BocN-MPO) (above) and the respective deprotected copolymer (below) with the signal used to estimate comonomer incorporation highlighted in red.

In the case of poly(LA-co-Bn-MPO) the intensity of the aromatic signals at δ = 7.35 ppm resulting from the introduced benzyl ester groups relative to the LA methine signal (δ = 5.17) was employed to determine the amount of incorporation. In the same way the relative intensity of the signal of the BOC protection group at δ = 1.45 ppm served as means of quantifying the incorporation of BocN-MPO. ^1^H-NMR spectra of copolymers (as well as deprotected copolymers) are shown in Figure 4 and Figure 5.

**Figure 6:**
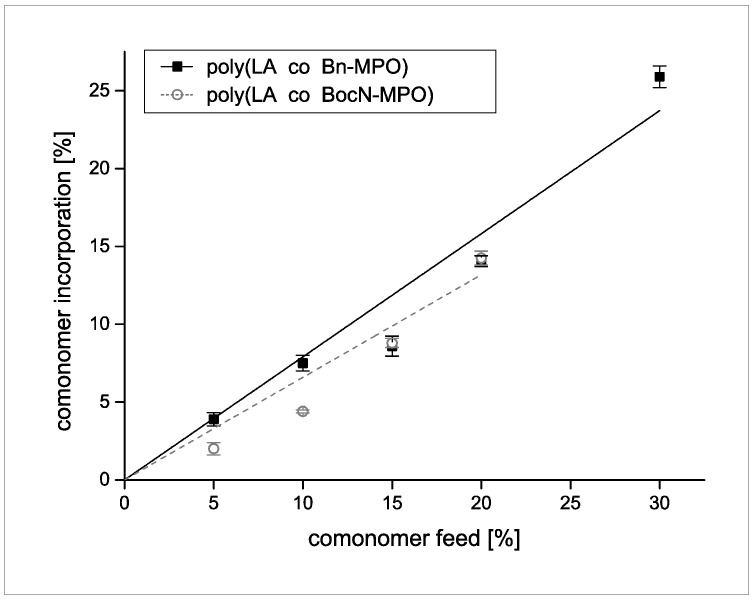
Incorporation efficiency of Bn-MPO and BocN-MPO into copolymers with LA.

Incorporation efficiencies as a function of comonomer feed are depicted in Figure 6. Maximum incorporation of 25% was achieved for the Bn-MPO comonomer by employing a comonomer feed of 30%. For poly(LA-co-BocN-MPO), a comonomer feed of 20% led to a maximum incorporation of 15%.

### 2.4. Deprotection of Copolymers

Charged or chargeable functional groups along the backbone of a polymer represent an interesting way to customize this material’s properties such as hydrophilicity/phobicity of polymeric surfaces or the surface charge of polymeric nanoparticles. The polymers presented here are amenable to deprotection reactions yielding PLA with pendant carboxylic acid and amine groups. Cleavage of the benzyl ester protecting groups (poly(LA-co-Bn-MPO)) was carried out by hydrogenation applying palladium catalysis. Deprotection of the amine groups in poly(LA-co-BocN-MPO) could be achieved using the strong acid TFA under water-free conditions. Advancement of the deprotection reactions was evaluated via ^1^H-NMR spectroscopy by examining the disappearance of the peaks allocated to the protecting groups (*cf*. Figure 4 and Figure 5). Complete removal of the benzyl ester groups was achieved after 48 h whereas the BOC-groups were cleaved after 1.5 h.

In the former case no degradation of the polymer chain was observed, with hydrogenation being a very mild deprotection reaction. The slightly lower M_n_ of around 5% its initial value was, within the range of inaccuracies, attributed to the loss of the protecting groups. However, treatment with TFA led to a decrease in M_n_ of the copolymers of around 50%. Losses of molecular weight in this magnitude will under certain circumstances be unacceptable. Yet, though meticulously executed under water free conditions, the effect of the strong acid on the polymer chains could not be eliminated. Therefore, alternative protecting groups such as the carboxybenzyl (Cbz) group that can be cleaved under milder conditions are called for and are currently under investigation in our group.

### 2.5. Thermal Properties of Copolymers

In order to determine whether the incorporation of the comonomers has an effect on material properties directly influencing the stability and shelf life of the copolymers, thermal behavior of the materials was investigated. Thermogravimetric measurements revealed decomposition temperatures (T_d_) of the copolymers between 240 °C and 260 °C, whereas a pure PLLA synthesized under analog conditions and with comparable molecular weight decomposed around 255 °C. The only minor differences in the values of T_d_ indicated that the incorporation of Bn-MPO and BocN-MPO did not lead to an impaired thermal stability as compared to the homopolymer.

**Figure 7:**
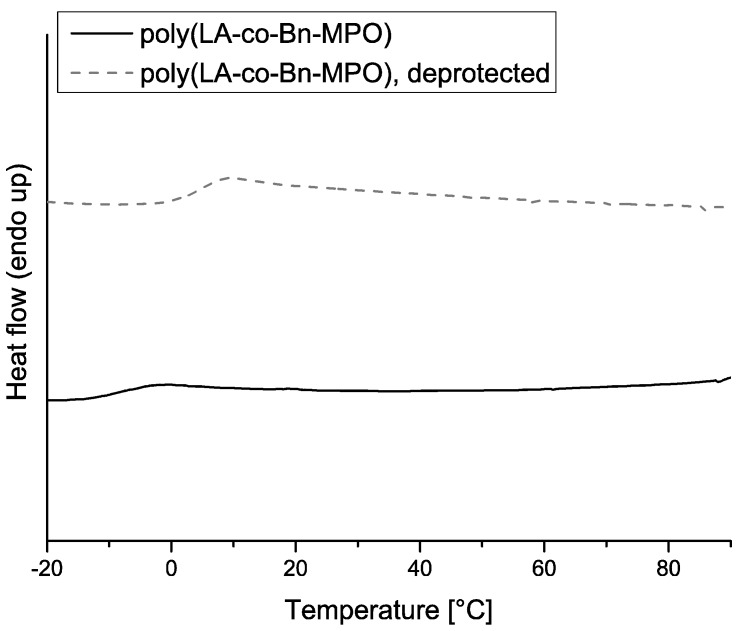
DSC curves for poly(LA-co-Bn-MPO) and deprotected poly(LA-co-Bn-MPO) with a comonomer incorporation of 14.1%.

**Figure 8:**
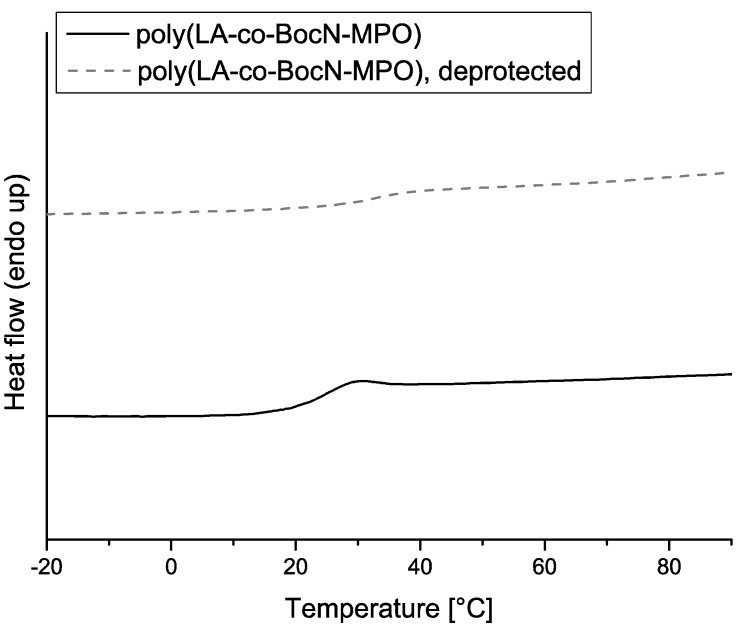
DSC curves for poly(LA-co-BocN-MPO) and deprotected poly(LA-co-BocN-MPO) with a comonomer incorporation of 9.9%.

DSC measurements were carried out to yield information on glass transition temperatures (T_g_) of the copolymers (*cf.* Table 1). The poly(LA-co-BnMPO) materials exhibited glass transition temperatures ranging from 7 to −11 °C, depending on the amount of incorporated comonomer. The copolymers derived from BocMAE‑MPO showed values of Tg in the range of 15 to 23 °C. In both cases this was a distinct decrease when compared to the T_g_ of pure PLA with similar M_n_ which was 54 °C. This behavior is supposed to result from the presence of pendant groups, leading to a plasticizer effect by increasing the interchain distances. Evolution of the T_g_ with increasing incorporation of comonomers is as expected for poly(LA-co-BnMPO), showing a decrease with increasing number of pendant groups. Interestingly, the poly(LA-co-BocN-MPO) materials showed the opposite trend of a rise in T_g_ with increasing comonomer incoroporation despite the degression in molecular weight. Removal of the protecting groups led to slight elevations in the glass transition temperatures of approximately 10 °C for all copolymers. The reduction in the size of the pendant groups might be the explanation for this observation. Furthermore, the absence of a second transition temperature in the case of all copolymers strongly suggests a random incorporation of the copolymers into the polymer backbone resulting in no significant blockiness. This observation is consistent with previous findings on the reactivity of a similar copolymerization system [28]. Exemplary DSC curves for copolymers and deprotected copolymers are shown in Figure 7 and Figure 8.

**Table 1:** Thermal properties of synthesized (co)polymers.

	M_n_ g·mol^−1^	Comonomer Incorp. [%]	T_d_ [°C]	T_g_ [°C]	T_g, after deprotection_ [°C]
**PLA**	9500	-	252	54	-
**poly(LA-co-Bn-MPO)**	8500	7.5	251	7	14
	9000	8.6	252	4	13
	10,000	14.1	241	−9	5
	3500	25.9	240	−11	0
**poly(LA-co-BocN-MPO)**	9500	7.7	262	15	23
	8000	9.9	257	22	31
	5000	15.7	254	23	31

All values of T_g_ observed for the copolymers were below physiological temperature which might prove valuable for conceivable applications of the materials such as soft nanoparticular formulations with a tunable particle-cell membrane interaction for transcellular delivery of active agents due to changes in material viscosity (and particle shape) [33].

## 3. Experimental Section

### 3.1. Materials

(3*S*)-*cis*-3,6-Dimethyl-1,4-dioxane-2,5-dione (L-lactide, 98%) was purchased from Sigma-Aldrich (St. Louis, MO, USA) and recrystallized from freshly distilled ethyl acetate (>99.5%, Roth, Karlsruhe, Germany) prior to use. Dichloromethane (p.a., Sigma-Aldrich) and trifluoroacetic acid (99%, Sigma-Aldrich) were distilled from P_2_O_5_ (puriss., Sigma-Aldrich) to eliminate water. 2-(Methylamino)ethanol (≥98%), 2-methyl-4-pentenoic acid (≥98%), acetone (p.a.), benzyl alcohol (≥99%), di-*tert*-butyl dicarbonate (97%), ethanol (p.a.), Oxone^®^, palladium on carbon (puriss., 10% Pd base), sodium phosphate dibasic dehydrate (p.a.) and tin(II) 2-ethylhexanoate (95%) were purchased from Sigma-Aldrich and used as received. 4-(dimethylamino)pyridine (≥99%) and *N*,*N*′-dicyclohexylcarbodiimide (DCC) (≥99%) were from Fluka Analytical (Buchs, Switzerland) and used without further treatment. Further products were chloroform-d (99.8% D, Deutero GmbH, Kastellaun, Germany), diethyl ether (≥99.7%, VWR International GmbH, Darmstadt, Germany), isohexanes (Ölfabrik Lahr, Lahr, Germany), Na_2_SO_4_ (p.a., AppliChem GmbH, Darmstadt, Germany) and silica gel (40–63 μm, Merck KGaA, Darmstadt, Germany) (all used as received).

### 3.2. Synthetic Procedures

#### 3.2.1. Synthesis of Benzyl 2-methylpent-4-enoate (Bn-MP)

DCC (36.2 g, 175 mmol, 1.00 eq.) was dissolved in dichloromethane (300 mL) and cooled to 0 °C. 4‑(Dimethylamino)pyridine (DMAP, 1.20 g, 9.82 mmol, 6 mol %), solutions of benzyl alcohol (22.7 g, 210 mmol, 1.20 eq.) in dichloromethane (30 mL) and 2-methyl-4-pentenoic acid (20.0 g, 175 mmol, 1.00 eq.) in dichloromethane (30 mL) were added. The reaction mixture was stirred at 0 °C for 3 h and at room temperature for an additional 12 h. The precipitate was filtered off and washed with dichloromethane (2 × 30 mL). Evaporation of the solvent *in vacuo* afforded the title compound as a light yellow oil (35.4 g, 173 mmol, 99%). ^1^H-NMR (300 MHz, CDCl_3_, 300K): δ (ppm) = 1.18 (d, 3H, C*H*_3_), 2.32 (dddd, 2H, CH_2_CHC*H*_2_CH), 2.59 (tq, 1H, CH_2_C*H*CH_3_), 5.04 (m, 2H, C*H*_2_CHCH_2_), 5.12 (s, 2H, OC*H*_2_Ph), 5.74 (ddt, 1H, CH_2_C*H*CH_2_), 7.35 (m, 5H, arom. *H*).

#### 3.2.2. Synthesis of Benzyl 2-methyl-3-(oxiran-2-yl)propanoate) (Bn-MPO)

Bn-MP (39.30 g, 192.4 mmol, 1.00 eq.) was dissolved in acetone (450 mL) and an aqueous sodium phosphate buffer solution (450 mL, 0.2 mol·L^‑1^, pH = 7.0) was added. An aqueous solution of Oxone (133.4 g, 432.9 mmol, 2.25 eq., in deion. H_2_O, 500 mL, pH = 7) was added dropwise over a period of 2 h at 0 °C while maintaining the pH of the reaction mixture in the range of 7.25–7.50 via addition of aqueous NaOH-solution (2 M). After completed addition of Oxone, the pH was controlled until no further decrease was observed. The mixture was allowed to warm to room temperature and stirring was continued for 12 h. The precipitated salts were filtered off and acetone was removed from the filtrate under reduced pressure followed by extraction of the resulting aqueous phase with dichloromethane (8 × 35 mL). The combined organic phases were washed with brine (2 × 30 mL), dried over Na_2_SO_4_ and the solvent was removed *in vacuo*. Column chromatography (ethyl acetate and isohexanes as eluent) afforded Bn-MPO as a pale yellow oil (21.70 g, 98.5 mmol, 51%). Unconverted Bn-MP could be re-isolated. ^1^H-NMR (300 MHz, CDCl_3_, 300 K): δ (ppm) = 1.27 (dd, 3H, C*H*_3_), 1.81 (m, 2H, CH_2_(O)CHC*H*_2_CH), 2.44 (m, 1H, CH_2_(O)C*H*CH_3_), 2.72 (m, 2H, C*H*_2_(O)CH), 2.94 (m, 1H, CH_2_(O)C*H*), 5.14 (s, 2H, OC*H*_2_Ph), 7.35 (m, 5H, arom. *H*). EA: C 70.87% (70.98%), H 7.44% (7.32%).

#### 3.2.3. Synthesis of *tert*-Butyl (2-hydroxyethyl)(methyl)carbamate (BocN)

A solution of 2-(methylamino)ethanol (9.01 g, 120 mmol, 1.20 eq.) in dichloromethane (50 mL) was cooled to 0 °C followed by dropwise addition of a solution of di-*tert*-butyl dicarbonate (21.83 g, 100.00 mmol, 1.00 eq.) in dichloromethane (40 mL) over a period of 1 h. The reaction mixture was stirred overnight at room temperature. After removal of the solvent under reduced pressure, brine (35 mL) was added and the resulting aqueous phase was extracted with ethyl acetate (5 × 30 mL). The collected organic layers were washed with brine (2 × 30 mL) and dried over MgSO_4_. Removal of the solvent under reduced pressure afforded BocN-MP as a pale yellow oil (17.00 g, 97.02 mmol, 97%). ^1^H-NMR (300 MHz, CDCl_3_, 300 K): δ (ppm) = 1.45 (s, 9H, C(C*H*_3_)_3_), 2.91 (s, 3H, NC*H*_3_), 3.38 (t, 2H, HOCH_2_C*H*_2_N), 3.73 (t, 2H, HOC*H*_2_CH_2_N).

#### 3.2.4. Synthesis of 2-((*tert*-Butoxycarbonyl)(methyl)amino)ethyl 2-methylpent-4-enoate (BocN-MP)

DCC (20.0 g, 97.0 mmol, 1.00 eq.) was dissolved in dichloromethane (250 mL) and cooled to 0 °C. DMAP (0.7 g, 5.7 mmol, 6 mol %), solutions of BocN (17.0 g, 97.0 mmol, 1.00 eq.) in dichloromethane (30 mL) and 2‑methyl-4‑pentenoic acid (11.1 g, 97.0 mmol, 1.00 eq.) in dichloromethane (30 mL) were added. The reaction mixture was stirred at 0 °C for 3 h and at room temperature for an additional 12 h. The precipitate was filtered off and washed with dichloromethane (3 × 30 mL). Evaporation of the solvent *in vacuo* afforded BocN-MP as a pale yellow oil (25.3 g, 93.2 mmol, 96%). ^1^H-NMR (300 MHz, CDCl_3_, 300 K): δ (ppm) = 1.16 (d, 3H, CHC*H*_3_), 1.46 (s, 9H, C(C*H*_3_)_3_), 2.31 (dddd, 2H, CH_2_CHC*H*_2_CH), 2.52 (tq, 1H, CH_2_C*H*CH_3_), 2.90 (s, 3H, NC*H*_3_), 3.46 (br s, 2H, C(O)OCH_2_C*H*_2_N), 4.17 (br s, 2H, C(O)OC*H*_2_CH_2_N), 5.05 (m, 2H, C*H*_2_CHCH_2_), 5.74 (ddt, 1H, CH_2_C*H*CH_2_).

#### 3.2.5. Synthesis of 2-((*tert*-Butoxycarbonyl)(methyl)amino)ethyl 2-methyl-3-(oxiran-2-yl)propanoate (BocN-MPO)

BocN-MP (25.0 g, 92.1 mmol, 1.00 eq.) was dissolved in acetone (450 mL) and an aqueous sodium phosphate buffer solution (450 mL, 0.2 mol·L^−1^, pH = 7.0) was added. An aqueous solution of Oxone (63.80 g, 207.6 mmol, 2.25 eq., in deion. H_2_O, 500 mL, pH = 7) was added dropwise over a period of 2 h at 0 °C while maintaining the pH of the reaction mixture in the range of 7.25–7.50 via addition of aqueous NaOH-solution (2M). After completed addition of Oxone, the pH was controlled until no further decrease was observed. The mixture was allowed to warm to room temperature and stirring was continued for 12 h. The precipitated salts were filtered off and acetone was removed from the filtrate under reduced pressure followed by extraction of the resulting aqueous phase with dichloromethane (8 × 35 mL). The combined organic phases were washed with brine (2 × 30 mL), dried over Na_2_SO_4_ and the solvent was removed *in vacuo*. Column chromatography (ethyl acetate and isohexanes as eluent) afforded Bn-MPO as a pale yellow oil (12.7 g, 44.1 mmol, 51%). Unconverted BocN-MP could be re-isolated. ^1^H-NMR (300 MHz, CDCl_3_, 300 K): δ (ppm) = 1.25 (dd, 3H, CHC*H*_3_), 1.45 (s, 9H, C(C*H*_3_)_3_), 1.78 (m, 2H, CH_2_CHC*H*_2_CH), 2.46 (m, 1H, CH_2_C*H*CH_3_), 2.72 (m, 2H, C*H*_2_(O)CH), 2.91 (s, 3H, NC*H*_3_), 2.95 (m, 1H, CH_2_(O)C*H*), 3.47 (br s, 2H, C(O)OCH_2_C*H*_2_N), 4.20 (br s, 2H, C(O)OC*H*_2_CH_2_N). EA: C 58.43% (58.52%), H 8.91% (8.77%), N 4.77% (4.87%).

#### 3.2.6. Copolymerization of L-Lactide with Bn-MPO and BocN-MPO

In a typical procedure, a total amount 30 mmol of pre-dried monomers (L-lactide with BnMPO or BocN-MPO) were added in the calculated ratio to a dry round bottom flask equipped with a magnetic stir bar and a rubber septum. After flushing with argon, the initiating mixture consisting of tin(II) 2-ethylhexanoate (0.7 mol %) and benzyl alcohol (1.4 mol %) in a small amount of DCM was added. The reaction mixture was placed in an oil bath at 90 °C to start the polymerization which was pursued for 12 h. The polymerization was terminated with ethanol by adding 1.0 mL of a 1:1(v/v)-mixture of DCM and ethanol. The resulting polymer was diluted with 5.0 mL of DCM, precipitated in ice-cold Et_2_O and dried *in vacuo* at room temperature to constant weight.

#### 3.2.7. Deprotection of Poly(LA-co-Bn-MPO)

In a round bottom flask equipped with a magnetic stir bar and a rubber septum, the polymer was dissolved in a 3:2 (v/v)-mixture of THF and MeOH. After addition of Pd/C (5 wt %, 10 wt % loading), the suspension was purged with hydrogen. The reaction mixture was vigorously stirred at room temperature under hydrogen pressure for 48 h. After filtration the deprotected polymer was obtained by precipitation in ice-cold Et_2_O and vacuum-dried at room temperature to constant weight.

#### 3.2.8. Deprotection of Poly(LA-co-BocN-MPO)

In a dried round bottom flask equipped with a magnetic stir bar and a rubber septum the polymer was dissolved in dry DCM and an excess of TFA was added to the solution. After stirring in an Argon atmosphere at room temperature for 1.5 h, the deprotected polymer was obtained by precipitation in ice-cold Et_2_O and vacuum-dried at room temperature to constant weight.

### 3.3. Methods

#### 3.3.1. Proton Nuclear Magnetic Resonance Spectroscopy (NMR)

Samples were measured on an ARX 300 MHz spectrometer (Bruker Corporation, Billerica, MA, USA) at 25 °C using CDCl_3_ as a solvent. Chemical shift in ppm was referred to the residual solvent peak (δ = 7.26 for CHCl_3_). Data analysis was carried out using TopSpin 3.0 software (Bruker).

#### 3.3.2. Gel Permeation Chromatography (GPC)

Analyses of molecular weight and polydispersity of the synthesized polymers were carried out applying a 1200 Series GPC-SEC analysis system consisting of pump G1310A, RI detector G1362A, and auto-sampler G1329A (Agilent Technologies, Santa Clara, CA, USA) which was equipped with SDVB columns (5 μm, 100 Å, 1000 Å, 10,000 Å) (PSS Polymer Standards Service GmbH, Mainz, Germany). Samples were prepared at a concentration of 4 mg·mL^−1^ in THF, filtered (0.45 μm filter) and eluted using THF at a flow rate of 1 mL·min^−1^ at 30 °C. Calibration was done using polystyrene EasiVial PS-H Tri-Pack with a nominal range of 162–6,000,000 g·mol^−1^ (Agilent Technologies). Elugrams were analyzed using WinGPC Unity, Build 5403 (Polymer Standard Solutions) according to the manufacturer’s guidelines and ISO 13885.

#### 3.3.3. Differential Scanning Calorimetry (DSC)

Thermal transitions were determined on a Pyris 1 differential scanning calorimeter (Perkin Elmer, Waltham, MA, USA). Polymer samples (approx. 15 mg) were sealed in aluminum pans and measured under a nitrogen atmosphere. Indium and cyclohexene were used for temperature calibration. Samples were subjected to a heat-cool-heat cycle ranging from −50 °C to 120 °C with a heating/cooling rate of 10 °C·min^−1^. Second heating cycle was used for data interpretation with Pyris Manager version 8.0.0.0172 (Perkin Elmer).

#### 3.3.4. Elemental Analysis

Elemental analyses were carried out on a VarioEL (Elementaranalysensysteme GmbH, Hanau, Germany) equipped with a thermal conductivity detector.

## 4. Conclusions

We have presented the synthesis of novel functional α,ω-epoxyesters as monomers for copolymerizations with cyclic lactones. With these monomers it was possible to obtain functionalized PLA with protected carboxylic acid and amine groups. Poly(LA-co-Bn-MPO) and poly(LA-co-BocN-MPO) copolymers with functionalizations up to 25% and 15%, respectively, could be synthesized, however, especially in the case of poly(LA-co-BocN-MPO) at the expense of molecular weights of the copolymers. Deprotection of the introduced functional groups yielded PLA copolymers with pendant carboxylic acid and amine groups that might prove valuable for applications as nanoparticulate delivery systems with hypothesized adaptable surface properties resulting from the introduction of functional groups with a potential for interaction and reactions with biologically active compounds. Future work will concentrate on overcoming the aspect of unsatisfactory molecular weights obtained at higher feed/incorporation and on exploiting the potential of these copolymers as materials for nanoparticle synthesis and coating applications.
